# Histone Modification in NSCLC: Molecular Mechanisms and Therapeutic Targets

**DOI:** 10.3390/ijms222111701

**Published:** 2021-10-28

**Authors:** Khuloud Bajbouj, Abeer Al-Ali, Rakhee K. Ramakrishnan, Maha Saber-Ayad, Qutayba Hamid

**Affiliations:** 1 College of Medicine, University of Sharjah, Sharjah 27272, United Arab Emirates; kbajbouj@sharjah.ac.ae (K.B.); rramakrishnan@sharjah.ac.ae (R.K.R.); qalheialy@sharjah.ac.ae (Q.H.); 2 Sharjah Institute for Medical Research, University of Sharjah, Sharjah 27272, United Arab Emirates; U20106060@sharjah.ac.ae; 3 Faculty of Medicine, Cairo University, Cairo 11559, Egypt; 4 Meakins-Christie Laboratories, Research Institute of the McGill University Health Center, Montreal, QC H4A 3J1, Canada

**Keywords:** histone deacetylase, demethylase, methyltransferase, KDM, LSD, tumour suppressor genes, vorinostat

## Abstract

Lung cancer is the leading cause of cancer mortality in both genders, with non-small cell lung cancer (NSCLC) accounting for about 85% of all lung cancers. At the time of diagnosis, the tumour is usually locally advanced or metastatic, shaping a poor disease outcome. NSCLC includes adenocarcinoma, squamous cell carcinoma, and large cell lung carcinoma. Searching for novel therapeutic targets is mandated due to the modest effect of platinum-based therapy as well as the targeted therapies developed in the last decade. The latter is mainly due to the lack of mutation detection in around half of all NSCLC cases. New therapeutic modalities are also required to enhance the effect of immunotherapy in NSCLC. Identifying the molecular signature of NSCLC subtypes, including genetics and epigenetic variation, is crucial for selecting the appropriate therapy or combination of therapies. Epigenetic dysregulation has a key role in the tumourigenicity, tumour heterogeneity, and tumour resistance to conventional anti-cancer therapy. Epigenomic modulation is a potential therapeutic strategy in NSCLC that was suggested a long time ago and recently starting to attract further attention. Histone acetylation and deacetylation are the most frequently studied patterns of epigenetic modification. Several histone deacetylase (HDAC) inhibitors (HDIs), such as vorinostat and panobinostat, have shown promise in preclinical and clinical investigations on NSCLC. However, further research on HDIs in NSCLC is needed to assess their anti-tumour impact. Another modification, histone methylation, is one of the most well recognized patterns of histone modification. It can either promote or inhibit transcription at different gene loci, thus playing a rather complex role in lung cancer. Some histone methylation modifiers have demonstrated altered activities, suggesting their oncogenic or tumour-suppressive roles. In this review, patterns of histone modifications in NSCLC will be discussed, focusing on the molecular mechanisms of epigenetic modifications in tumour progression and metastasis, as well as in developing drug resistance. Then, we will explore the therapeutic targets emerging from studying the NSCLC epigenome, referring to the completed and ongoing clinical trials on those medications.

## 1. Introduction

Lung cancer is the most common malignancy worldwide, with the highest mortality among all cancers. By 2030, it is estimated to kill 10 million people per year worldwide [1]. There are four fundamental histological types, which account for approximately 95% of all lung cancers. These include squamous cell carcinoma (SCC) (20–35%), adenocarcinoma (ADC) (30–50%), and large cell cancer (LCC) (9%), all of which belong to non-small cell lung carcinoma (NSCLC) (80–84%). Other NSCLC subtypes, such as carcinoma of adenosquamous and sarcomatoid subtypes, are much less common. Small cell carcinoma (SCLC) accounts for only 16–20% of all lung cancer [2]. Details on the pathology and classification of lung cancer were covered in several review articles [2,3]. Depending on the stage of the tumour, the five-year survival after lung cancer surgery ranges between 10 and 70% [4]. NSCLC in older age groups usually carries the lowest cancer-specific survival and the worst overall survival (OS) [5]. However, thanks to therapeutic advances, the two-year relative survival rate for NSCLC increased from 34% (in 2010) to 42% (in 2015), with an absolute rise of 5% to 6% for every stage of diagnosis [6]. While cigarette smoking is by far the leading cause of lung cancer in both men and women, other etiologic factors such as second-hand smoking, radon, asbestos, or heavy metals also play a role [7]. Interestingly, lung exposure to cigarette smoke may affect histone modifications. In addition, nickel, chromate, and arsenite in tobacco induce histone H3K9 dimethylation and stimulate histone deacetylation [8,9].

In the current review, we will first give an overview on the initiation and signalling pathways in NSCLC, focusing on the role of histone modification. The patterns and mechanisms of histone modification will be discussed. Then, the role of epigenetic modifying agents in NSCLC will be described in vitro, in pre-clinical trials, and then in clinical trials. We will also discuss the different combination therapy options in the treatment of NSCLC.

### Lung Cancer Initiation and Signalling Pathways

Rather than capitalizing on specific factors, genetic abnormalities correlated to lung cancer risk should be considered in the context of signalling pathways with distorted main functions. Several major pathways with key components have dysregulated functions in lung cancer, with emerging significance in terms of targeted therapy. Aberrations in the growth-stimulation signalling pathways or tumour suppressor gene (TSG) pathways significantly contribute to lung cancer development [10]. Oncogene mutations cause tumour cells to become “addicted” to their aberrant functions. Notably, the TSGs are linked to environmental factors such as smoking and air pollution [11].

Exposure to cigarette smoke can cause an inflammatory response in a smokers’ airways, releasing several inflammatory mediators and growth factors (e.g., transforming growth factor β (TGF-β), epidermal growth factor receptor (EGFR), interleukin-1 (IL-1), IL-8, and granulocyte colony stimulating factor (G-CSF)). Such inflammation can last for decades after the cessation of smoking, leading to pathological and neoplastic changes [12]. Interestingly, KRAS mutations are increased in smokers. On the contrary, EGFR and HER2 mutations are increased in non-smokers. Such an observation adds to the findings that lung cancer in smokers and non-smokers arises via distinct pathogenic pathways [10]. Hazardous air pollutants or chemicals released into the environment may have a negative impact on human health. Gases (including sulphur dioxide (SO_2_), nitrogen oxides (NOx), carbon monoxide (CO)), heavy metals, and particulate matter (PM) are all air pollutants that have a deleterious impact, particularly on the respiratory system. According to the International Agency for Research on Cancer (IARC), both outdoor and PM air pollutants are Group 1 carcinogens [13].

## 2. Epigenetics in Lung Cancer

Cancer is considered a genetic disease. Mutations are one of the cancer hallmarks. However, there is also mounting evidence that the epigenetic modification of gene expression (including the mutant genes) plays a key role in carcinogenesis of the lung and other cancers [14]. Epigenetics refer to heritable chromatin modifications that impact gene expression (among a myriad of DNA-dependent processes) without a direct effect on the coding sequence of the DNA [15]. Epigenetic regulation of gene expression occurs at three levels: DNA (DNA methylation), protein (histone/nucleosome modification), and non-coding RNA (ncRNAs). Amongst the major epigenetic regulatory mechanisms, DNA methylation is perhaps the most studied, and it is responsible for gene silencing. Intriguingly, histone proteins can be modified in a variety of ways, including acetylation, methylation, and phosphorylation, in addition to ubiquitylation and sumoylation. Histone covalent modifications, opposed to DNA methylation, not only silence the expression of specific genes, but rather promote transcription. Out beyond the classical epigenetic mechanisms, ncRNAs, particularly microRNAs (miRNA) and long ncRNAs (lncRNAs), are also recognized as epigenetic modifiers [16]. Mutations in epigenetic regulatory mechanisms and epigenetic pattern disruptions have been linked to a variety of tumour types, which include lung cancer via TSG silencing and oncogene activation. Chemotherapy resistance is also linked to epigenetic changes [15].

Recent epigenetic advances have improved our knowledge of the basic mechanisms of carcinogenesis. Changes in the epigenetic factors lead to alterations in the expression of key oncogenes and TSGs [17]. Several epigenetic processes in lung cancer have an impact on cancer hallmarks, such as proliferation, invasion, metastasis, apoptosis, and regulations of cell cycle [18]. Further to cancer hallmarks, epigenetic deregulation in lung cancer affects numerous impactful signalling pathways, including the ERK family, NF-kB signalling pathway, and Hedgehog signalling pathway [17]. Concurrently, epigenetic marks shed light on the identification of potential cancer biomarkers for early screening, monitoring, and therapeutic approaches of NSCLC.

**Figure 1:**
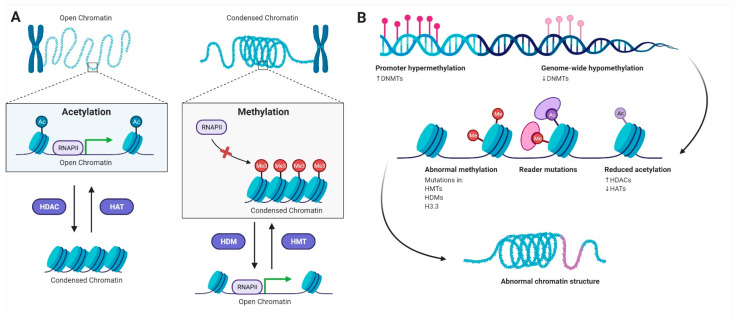
Epigenetic regulatory mechanisms linked to lung cancer. (**A**) Acetylation of histones reduces its positive charge, thereby relaxing the tightly coiled negatively charged DNA wrapped around them. The open chromatin structure enables access to the transcriptional machinery (including RNA polymerase and transcriptional factors), resulting in active gene transcription. Conversely, methylation of histones is generally associated with a condensed chromatin structure, which prevents access to the transcriptional machinery repressing gene transcription. While histone acetyltransferases (HATs) introduce acetyl groups onto lysine residues on histones, histone deacetylases (HDACs) carry out deacetylation of histones. Similarly, histone methyltransferases (HMTs) promote the addition of mono-, di-, or tri-methyl groups at arginine and/or lysine residues on histones, while histone demethylases (HDMs) are responsible for removing these methyl groups. (**B**) Cancer cells display hypermethylation of multiple promoter CpG islands as well as a genome-wide loss of DNA methylation at sporadic CpGs. Methyl-binding proteins are further capable of recruiting HDACs. The overexpression of HMTs and HDACs thus results in reduced chromatin access and silencing of TSG. This abnormal epigenetic activity paves the way to lung cancer development and progression.

In addition to genetic changes occurring in lung cancer, epigenetic modifications also impact various components of chromatin modifying events such as histone modifications, DNA methylation, and microRNA regulation [15]. Histone deacetylases (HDACs) are frequently overexpressed in cancers and have emerged as promising therapeutic targets [19]. HDAC overexpression can result in TSG silencing and abnormal transcription as a result of the modified expression and/or mutations of genes encoding histone acetyltransferase (HAT) or HDAC enzymes or their binding congeners, which are directly connected to carcinogenesis [20] (Figure 1).

### 2.1. Histone Modifications

The DNA is packed as chromatin in eukaryotic cells, with nucleosomes serving as functional units. Every other nucleosome is made up of an octamer of four core histones (H3, H4, H2A, and H2B) rolled up in 147 base pairs of DNA [18]. The nucleosome’s core is formed by the globular regions of histones, while the N-terminal tails protrude from the nucleosomes and are enhanced with a diverse array of posttranslational modifications (PTMs). Histone tails are altered by a large group of non-histone chromatin-associated proteins known as chromatin-modifying enzymes. These enzymes reside in cells as multicomponent protein complexes that are regularly recruited to chromatin in association with DNA-bound transcription factors [21]. Numerous covalent PTMs in histones and associated regions of DNA play critical roles in genomic functions by binding specific transcription factors and co-activators, altering the structural properties of chromatin [22].

Based on their functions, chromatin modification enzymes are divided into four groups: acetylation HATs, deacetylation by HDACs, methylation by HMTs, and demethylation by HDMs (Figure 1). The generated PTMs could function concertedly or alone to promote activation or suppression of chromatin-mediated gene expression of inflammatory cytokines, cell cycle arrest, senescence, apoptosis, growth factors, and antioxidants, in addition to TSGs associated with lung cancer [23].

### 2.2. Histone Modifications Cross Talk

Histones can be influenced by a myriad of PTMs, resulting in a plethora of combinatorial patterns. These can arise on any of the four core histones and can even differ between tails of the same histone within a single nucleosome or nucleosomes. Identifying which essential modifications influence tumourigenesis and which are bystanders is a critical complicated goal, since various interactions between PTMs occur (modification cross talk). In some cases, histone PTMs can function as exclusion marks, restricting the appearance of other marks [22]. Histone lysine acetylation and arginine methylation can both co-localize and initiate other methyltransferase actions. Furthermore, histone methylation and phosphorylation can regulate each other. An important example is when an adjacent phosphorylation event disrupts chromodomain recognition of a methylated lysine. Fundamentally, the challenge of definitively linking changes in primary chromatin to cancer and then identifying how cross talk contributes to cancer are critical questions to be addressed. Intriguingly, histone PTM around the DNA damage site is crucial in the process of DNA repair [21].

### 2.3. Histone Acetylation/Deacetylation

A number of histone modifiers and chromatin-bound proteins regulate transcription activation and repression. At the stable processes of the cell, an equilibrium between specific modifications and modifiers is sustained to retain chromatin structure, perform the precise gene expression program, and control the biological output (Figure 1). When the balance is changed, cell phenotypes can be altered, setting the stage for disease onset and progression [24]. Studying the main regulators of histone modifications will thus aid in the development of chemical probes to preserve homeostasis and regain the balanced state of the cell. Chromosomes are structured into coiled chromatin fibers, made up of units (nucleosomes), which are a central core of histone proteins, around which the DNA is coiled [25]. This condensed structure renders the DNA transcriptionally inactive via decreasing the interaction between the DNA and its transcription and replication proteins. Inversely, chromatin composition can be reversibly altered by uncoiling to grant access to DNA-binding factors and activate transcription. Histone acetylation is a major remodelling process, in which HATs introduce acetyl groups to lysine residues on histone N-terminal tails that protrude from the nucleosome complex [21]. Histone acetylation results in a loss of positive charge, decreased affinity between histones and DNA, and resultant uncoiling to give access to RNA polymerase and transcription factors. HDACs, on the other hand, eliminate acetyl groups and operate as a transcriptional suppressor [15].

Histone deacetylation is modulated by DNA methylation via repressor protein binding to methylated CpGs in DNA. As a result, PTMs of histones via acetylation and deacetylation are a main determinant of transcription regulation, impacting gene expression. At least 18 human HDAC enzymes have been identified [26]. Based on their homology to yeast HDACs, subcellular localization, tissue specificity, and enzymatic activities, they are categorized into four classes (I, II, III, and IV) [27]. The classical HDAC family is made up of class I (HDAC1, 2, 3, and 8) and class II (HDAC4, 5, 6, 7, 9, and 10) HDACs, with HDAC11 exhibiting interlaced properties. Class I HDACs are found in the nucleus, while class II HDACs travel to and from the nucleus. HDACs are actively engaged in a variety of biological processes, such as cell cycling, proliferation, differentiation, cell death, DNA replication, mitosis, and cancer development [26]. Interestingly, HDAC inhibition was recognized through empirical screening of some agents that were shown to induce cancer cell differentiation. These agents, such as butyrate, trichostatin A (TSA), and suberoylanilide hydroxamic acid (SAHA) (vorinostat) were discovered to have HDAC inhibitory action [28]. Three decades ago, Breslow et al. described SAHA to arrest the growth of murine erythroleukemia cells at low concentrations, positioning it as a lead HDAC inhibitor (HDI) [29].

The HDIs have the ability to kill cancer cells through apoptosis, autophagy, necrosis, reactive oxygen species (ROS), cell cycle arrest, tumour angiogenesis suppression, and immunomodulatory effects. They stimulate the death receptor, as well as intrinsic mitochondrial pathways, reducing the overall apoptotic threshold of tumour cells. They increase the expression of death receptor pathway-related pro-apoptotic genes (TRAIL and DR5) and/or the intrinsic apoptotic pathway (Bax, Bak, and APAF1). On the other hand, HDIs decrease the expression of pro-survival genes (BCL-2 and XIAP). They also selectively induce BH3-only proteins, resulting in the activation of the intrinsic apoptotic pathway [30]. Aside from their direct anti-cancer effects, HDIs boost the immune system by increasing the expression of the major histocompatibility complex (MHC) class I and II proteins, as well as costimulatory and/or adhesion molecules such as CD80, CD86, human leukocyte antigen (HLA)-DR, HLA-ABC, and intracellular adhesion molecule-1 (ICAM-1,28). HDIs could also improve immune responses by modifying immune cell activities, either directly or indirectly via the cytokine secretion modulation [28]. Both in vitro and in vivo studies have showed that HDIs suppress the growth of a wide spectrum of cancer cells, including those of lung cancer [15].

### 2.4. Histone Methylation

Two important enzyme groups control histone methylation: methyltransferases and demethylases. Histone methylation mainly involves the lysine (K) and arginine (R) residues on the N-terminal of the histone tail [31]. Histone methylation can promote or inhibit various gene transcriptions, denoting an intricate function in cancer, based on the location and methylation level of the target amino acid. Histone H3 has five lysines amenable for modification by methylation (K4, K9, K27, K36, and K79). Similarly, K20 of histone H4 undergoes methylation by a specific histone lysine methyltransferase. H3K4 and H3K36 methylation can activate gene transcription, whereas H3K9, H3K27, H3K79, and H4K20 methylation can suppress gene transcription [32]. Abnormalities in histone methylation are closely linked to various cancers.

**Figure 2:**
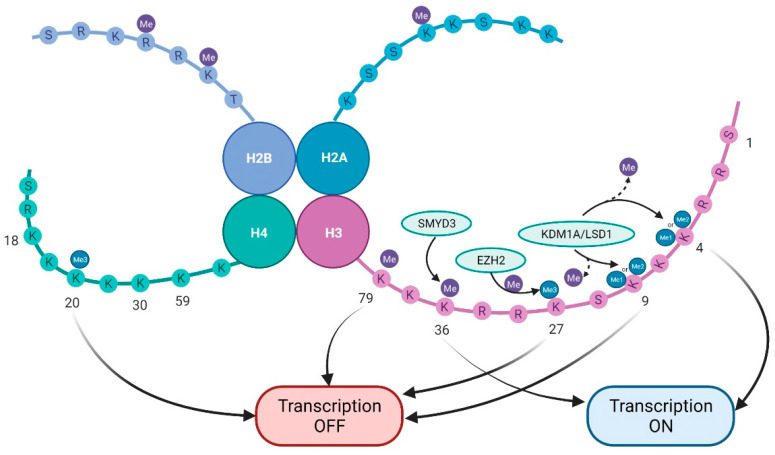
Histone methylation landscape in lung cancer. The lysines K4, K9, K27, K36, and K79 on histone H3 and K20 of histone H4 are amenable for modification by methylation. While H3K4 and H3K36 methylation activate gene transcription, H3K9, H3K27, H3K79, and H4K20 methylation suppress gene transcription. Histone lysine methyltransferases (KMTs) add methyl groups to histones and are ‘writers’ of the histone code. SMYD3 targets H3K36, and EZH2 mediates trivalent methylation of H3K27, causing chromatin condensation and subsequent repression of TSG transcription. On the other hand, demethylases, such as KDMs, are known as ‘erasers’ of methyl groups, and consequently, KDM1A/LSD1 targets H3K4me2/me1 and H3K9me2/me1.

The role of histone lysine methyltransferases (KMTs) is to add methyl groups. Thus, they function as ‘writers’ of the histone code, whereas histone lysine demethylases (KDMs) are known as ‘erasers’ of methyl groups (Figure 2) [33]. Catalyzed by methyltransferase, methylation leads to adding one, two, or three methyl groups. The trivalent methylation is known as hypermethylation [34]. For instance, KDM1A (also known as lysine specific demethylase, or LSD1) targets H3K4me2/me1 and H3K9me2/me1 and is overexpressed in NSCLC [35]. EZH2 mediates trivalent methylation of lysine 27 on histone H3 (H3K27me3), thus causing condensation of chromatin and repression of TSG transcription [36]. SMYD3 targets H3K36 and plays a pivotal role in the regulation of oncogenic Ras signalling in NSCLC (and also in pancreatic ductal adenocarcinoma) [37]. In a bioinformatics-based study in 2019, Li et al. analyzed gene variants, mRNA expression of histone methyltransferases, and demethylases in NSCLC in relation to the patients’ data. They suggested that some genes related to histone methylation may have potential prognostic and/or therapeutic value, awaiting further biological validation [38].

### 2.5. The Histone Code of Lung Cancer

Histone deacetylases and histone methyltransferases are linked to methyl-CpG binding proteins and DNA methyltransferases. Human cancers have a tremendous overall loss of DNA methylation, but they develop specialized patterns of hypermethylation of particular promoters. DNA methylation tends to occur alongside other epigenetic modifications. The acetylation and methylation condition of particular lysine residues within the tail of nucleosome core histones play a significant role in modulating chromatin packaging, nuclear structure, gene expression, and genomic stability [39]. The process of promoter CpG island hypermethylation of transcriptionally repressed TSGs is affiliated with a specific combination of histone markers in lung cancer cells. Examples include deacetylation of histones H3 and H4, the loss of histone H3 lysine 4 trimethylation, and the gain of H3K9 and H3K27 trimethylation.

The epigenetic landscape of cancer cells that are fundamentally distorted in appearance, when contrasted to normal cells, has demonstrated the effect of histone code alterations in lung cancer tumourigenesis and prognosis. In NSCLC and pre-invasive bronchial dysplastic lesions, there is an increased acetylation of H4K5/H4K8 and loss of trimethylation of H4K20. H4K20 trimethylation loss was found to be associated with a subpopulation of early stage I ADC with shortened survival [40]. Furthermore, H2 and H3 acetylation and trimethylation states in NSCLC enable the identification of prognostic markers. The prognostic value of epigenetic alterations involving multiple histones, particularly H2A (H2AK5ac) and H3 (H3K4me2, H3K9ac), was higher in early NSCLC, and evaluating these changes may aid in the selection of early-stage NSCLC patients for adjuvant therapy [41]. Likewise, H3K4me2 and H3K18ac cellular levels were relatively low, and the identified histone modification trends were independent predictors of prognosis in ADC [42]. These findings strongly suggest that reduced cellular levels of specific histone modifications are likely to predict a shorter survival time.

Intriguingly, histone modification levels positively correlated with one another; loss of one histone modification generally correlated with loss of other modifications within a patient [42]. Furthermore, demethylated DNA repetitious elements in cancer DNA may also be demethylated and/or deacetylated on their associated histones. The biological consequences of these histone modifications at repeated elements are unknown, but they are likely affiliated with a much more aggressive phenotype [42]. In general, a higher frequency of cancer cells, with reduced global levels of histone modifications, signifies a poorer clinical outcome, i.e., a higher risk of tumour relapse and/or a shorter survival time in lung cancer [43]. The modalities influencing histone alterations are still being investigated and may be ascribed to incorrect targeting, distorted expression, and/or activity of histone-modifying enzymes due to genetic mutations, expression shifts, and/or posttranslational control [44]. Modern innovations employing genome-wide cancer epigenetics methodologies have revealed that global changes in histone modification patterns correlate with DNA methylation in lung cancer [45].

### 2.6. Histone Deacetylase Expression in Lung Cancer

HDAC expression in lung cancer tissue samples has been investigated in a number of studies. In a study of 102 NSCLC resection specimens, HDAC1 mRNA and protein expression were higher in patients with stages III and IV lung cancer, as opposed to stage I or II. There was no significant difference in mRNA expression between tumour and non-tumour lung tissue [46]. The mRNA expression of HDAC1 to 8 and 10 was linked with prognosis in a study of 72 NSCLC specimens [47]. Low expression of class II HDACs, particularly HDAC10, was linked with a worse prognosis after surgery. HDAC3 protein expression was observed to be upregulated in SCC specimens employing antibody arrays and was verified by immunoblot analysis [48]. These preliminary studies suggest that HDACs suppress pivotal gene pathways implicated in protection against lung cancer.

## 3. Lung Cancer Therapeutics

Surgery, chemotherapy (platinum-based regimens), radiotherapy, combined chemo-radiotherapy, and targeted therapy, either alone or in combination, are conventional therapies for lung cancer. Surgery to extract the tumour and nearby lymph nodes is the most consistent and effective treatment in the early disease. When surgery is not an ideal solution, radiotherapy and/or chemotherapy may be recommended [49]. As previously mentioned, the two-year relative survival rate for NSCLC has gained an absolute rise of 5–6% over 5 years, thanks to advances in therapeutic modalities for late stages, including targeted EGFR tyrosine kinase inhibitors (TKIs) and, to a lesser extent, immunotherapy [6]. The survival improvement in earlier stages of the disease is likely due to the implementation of advanced surgical procedures (e.g., video-assisted thoracoscopic surgery) [50].

Epigenetically active drugs may aid two groups of lung cancer patients in particular: those who are not suitable for aggressive chemotherapy and those with high-risk NSCLC. The first group, i.e., patients who are ineligible for chemotherapy, could still be qualified for epigenetic therapy, which typically has a safer profile. Epigenetic therapies, instead of being cytotoxic like traditional chemotherapeutics, are believed to promote apoptosis and/or differentiation by overturning aberrant gene silencing or activation. In theory, this might eradicate only cancer cells while leaving normal cells alone, leading to fewer and less severe side effects [51]. The second group consists of high-risk NSCLC patients, defined as those with a shortened relapse-free survival (RFS) who appear to be predisposed to relevant epigenetic alterations, such as abnormal DNA methylation of HIST1H4F, PCDHGB6, NPBWR1, ALX1, and HOXA9 [52].

### 3.1. Histone Modifications in Lung Therapeutics

#### 3.1.1. Histone Deacetylases Inhibitors in Preclinical Studies

For decades, numerous HDIs have been produced and studied for their possibility as anti-cancer agents, ever since the identification that TSA inhibited HDACs and triggered differentiation and cell cycle arrest in mammalian cells [53]. There are several classes of HDIs, including hydroxamate, cyclic peptide, aliphatic acid, and benzamide classes [26]. Aiming at a nuclear-specific class I HDAC could have a more specific effect on histone acetylation with minimal side effects on non-histone cytoplasmic proteins. HDI treatment of cancer cells induced G1-phase cell cycle arrest by activating p21 and suppressing cyclin expression. Such agents have demonstrated the ability to activate both extrinsic and intrinsic apoptotic pathways by changing the expression of death receptors and ligands, in addition to the balance of major intracellular pro-apoptotic and antiapoptotic regulators [54,55]. Preclinical studies in NSCLC cell lines have illustrated that initiation of apoptosis in response to HDIs also entails checkpoint kinase 1 downregulation [56]. Furthermore, HDIs also cause DNA damage, downregulate the expression of DNA repair genes, repress proangiogenic and matrix remodelling genes, and disrupt glucose metabolism by targeting the glucose transporter 1 and hexokinase 1. Ultimately, HDIs may regulate cancer cell immunogenicity by upregulating molecules participating in T-cell and natural killer cell activation, such as MHC class I and II, CD80/CD86, and MHC class I chain-related molecules (MICA/MICB) [55].

Miyanaga et al. tested 16 NSCLC cell lines with HDAC inhibitors, including TSA and vorinostat, and found that both had anti-tumour activity in 50% of the NSCLC cell lines [28]. As a result, HDIs may exert their anti-cancer action, at least in part, by decreasing tumour cell responsiveness to TNF-mediated activation of the NF-κB pathway, as TNF-receptor-1 expression and TNF-mediated NF-κB nuclear translocation were all reduced in HDI-treated NSCLC cells [57].

Several chromatin remodelling studies suggest that HDIs have biological implications in lung cancer cell lines. TSA inhibited telomerase activity albeit with no effect in NSCLC cell lines [58]. Treatment with the HDI sodium butyrate caused global histone hyperacetylation and chromatin decondensation in the A549 cell line [59]. TSA treatment of the H69 SCLC cell line resulted in a chromatin decondensation pattern of nuclear texture [60].

Pathways that promote the responsiveness of lung cancer cells to HDIs have been previously investigated. When tested in A549, PC14, FK228, a lung ADC cell line that is remarkably more resilient, HDIs blocked the Akt-mediated signalling pathway in the A549 cells but not in the resistant PC14 cells [61]. In another study of A549 cells, TSA was found to be pro-apoptotic, as evidenced by the downregulation of the antiapoptotic Bcl-2 protein, the upregulation of the pro-apoptotic Bax protein, and caspase activation [62]. On the other hand, in an analysis of the SCLC cell lines H69 and H526, the HDI FR901228 prompted caspase-dependent apoptosis via the mitochondrial pathway instead of the death receptor pathway [63].

HDAC inhibition causes acetylation not only of histones but also transcription factors such as p53, GATA-1, and estrogen receptor-alpha [64]. TSA-induced acetylation of histones H3 and H4 in lung cancer cells resulted in the re-expression of a number of TSGs, such as TGFBR2, SATB1, C/EBP alpha, MYO18B, and DAPK [65]. Zhong et al. discovered over 200 genes highly expressed by TSA treatment, using high-throughput gene expression microarrays coupled with pharmacologic inhibition of DNA methylation and histone deacetylation in NSCLC [66]. Some of these genes (NRIP3, CYLD, CD9, ATF3, and OXTR) verify the role of histone deacetylation in their silencing. More details on the effect of histone modification in lung cancer were covered in previous reviews [67].

HDIs (e.g., TSA and SAHA) exert a number of histone- and HDAC-independent functions [68]. In addition, the link of TSA to inflammatory pathways is intricate. TSA treatment was reported to upregulate cyclooxygenase-2 (COX-2) and CXCL12 in mouse macrophages and to downregulate proinflammatory genes encoding TNF-α, IL-12p40, IL-6, endothelin 1, and the chemokines CCL2/monocyte chemotactic protein-1 (MCP-1) and CCL17 [49]. These findings indicate that individual HDIs have interconnected mechanisms of action on both deacetylation and non-histone pathways, which mandate further evaluation.

#### 3.1.2. Histone Deacetylases Inhibitors (HDIs) in Clinical Use

Based on their structure, HDIs are classified into six groups [69]. A few HDIs have been shown to strengthen the cytotoxic effects of radiation by inhibiting DNA repair and enabling apoptosis in human NSCLC cells, having a significant synergism of action with conventional NSCLC chemotherapeutic agents [69]. Several HDIs are already being tested in clinical trials for the treatment of NSCLC patients.

Clinical data for HDI monotherapy in NSCLC are limited due to the small number of NSCLC patients in many trials. In particular, the outcomes in advanced, solid tumours have been underwhelming. While these agents are well tolerated, the best response is typically a stable disease, with rates ranging from 15 to 75% based on clinical context and the HDI evaluated [56]. An overview of clinical studies on HDIs as monotherapy and combinatorial therapy is presented in Table 1. While the existing information suggests that current HDIs are doubtful to supply a substantial benefit to NSCLC patients as monotherapy, they may be useful in combination with other agents. Vorinostat demonstrated the ability to enhance the response rate to first-line carboplatin and paclitaxel therapy in advanced NSCLC, but no survival benefit was observed [70]. In advanced chemo-refractory NSCLC, the introduction of entinostat to erlotinib presented no overall benefit compared to erlotinib alone, yet appeared to enhance survival in a subgroup of patients with high tumour E-cadherin levels at diagnosis [70,71].

#### 3.1.3. Suberoylanilide Hydroxamic Acid (SAHA, Vorinostat)

**Figure 3:**
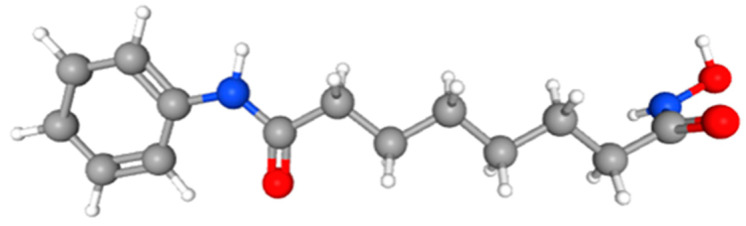
Chemical Structure of Vorinostat [92].

Vorinostat is a member of the hydroxamic acid group, which is the most diverse class of inhibitors with increased affinity for HDAC and has been shown to suppress both class I and II HDACs. At sub-micromolar concentrations, vorinostat prevents the enzymatic activity of HDAC1, HDAC2, and HDAC3 (class I) and HDAC6 (class II) [89]. Vorinostat (Figure 3) is a polar-planar compound of second generation that elicits cell arrest, differentiation, and/or apoptosis in a variety of transformed cells [90]. Vorinostat also demonstrated antiproliferative and pro-apoptotic effects in a variety of mouse xenografts and cancer cells, which include prostate, bladder, and breast carcinoma, as well as myeloma [91].

**Figure 4:**
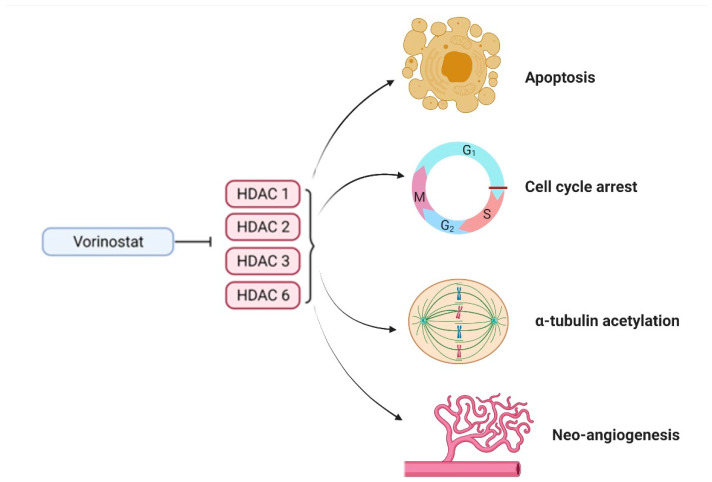
Multiple mechanisms of action of vorinostat in NSCLC.

Vorinostat can also initiate G1/G2 cell-cycle arrest in a cell-dependent and dose-dependent manner, as well as disrupt vascular endothelial growth factor (VEGF) signalling, implying that vorinostat might also hinder tumour neovascularization (Figure 4) [93]. Numerous studies on the action of vorinostat in conjunction with cytotoxic agents targeting chromatin DNA (such as etoposide, camptothecin, cisplatin, doxorubicin, 5-fluorouracil, and cyclophosphamide) have revealed synergistic and additive function in a wide range of cultured human transformed cell lines [94]. The effect of p53 gene condition on the interaction of vorinostat and carboplatin (a DNA targeting agent) across several NSCLC cell lines has been researched. Vorinostat increased carboplatin-induced cytotoxicity in NSCLC cells with wild-type p53, but not in cells lacking p53, implying the interference of a p53-dependent pathway. As a result, the addition of vorinostat may facilitate a decrease in the conventional carboplatin dose while optimising the overall therapeutic index [95].

Interestingly, the acetylation status of both tubulin and tubulin-associated proteins has been shown to regulate microtubule dynamics, implying that HDIs may interact favourably with taxanes to stop microtubule formation and initiate apoptosis [96]. HDAC6 exhibited the control alpha-tubulin deacetylation, implying that inhibiting HDAC6 with vorinostat might stabilize microtubules [96]. Because paclitaxel was shown to preferentially attach to stabilized microtubules, earlier administration of vorinostat may increase the effects of paclitaxel. Furthermore, the vorinostat ability to trigger G2/M cell-cycle accumulation and the increased sensitivity of G2/M cells to paclitaxel supports the idea that vorinostat may potentiate paclitaxel’s anti-tumour activity [97].

### 3.2. Modifiers of Histone Methylation

Inhibitors of methyl-transferases and demethylases exert anti-cancer effects against several solid tumours, including NSCLC [98]. KDM6A has a controversial role in NSCLC. It is a histone demethylase that antagonizes TGF-β induced epithelial–mesenchymal transition [99]. KDM6A loss leads to a better sensitivity to EZH2 inhibitors, denoting that patients with NSCLC with a specific histone methylation pattern may benefit from specific epigenetic modifying therapy [100]. Tumour heterogeneity represents a key phenomenon in most cancers. More than a decade ago, Sharma et al. consistently identified a small subset of reversibly “EGFR inhibitor-tolerant” cells, acquiring their resistance through an IGF-1-mediated signalling. Such resistance could be overcome through epigenetic manipulation, including pharmacological inhibition of the histone demethylase KDM5A/RBP2/JARID1A and reduced H3K4me2/me3 acetylation. Such “drug-tolerant cells” (later on known as cancer stem cells) with their distorted chromatin were extremely sensitive to the HDI effect [98].

A number of histone methylation inhibitors are under investigation in clinical trials [98,101]. Previous studies linked LSD1 aberrations with multiple malignancies, and the most promising results of LSD1 inhibitors were observed in acute myeloid leukaemia and SCLC [102], with GSK-2879552 and RG6016 representing a promising novel epigenetic approach for SCLC. Patients with NSCLC were considered a subset of recruited patients “with solid tumours” in those trials (e.g., NCT02082977).

### 3.3. Combination Therapy in Lung Cancer Using Histone Modifying Agents

#### 3.3.1. Preclinical Combination Therapy

Two new HDIs (ST2782 and ST3595) are synergized with taxanes, which operate by stabilizing microtubules in the spindle machinery and disrupting mitosis. In different cancer cell lines, including the NSCLC cells H460 and A549, combination therapy was preceded by an escalation in growth inhibition, apoptosis, and cell cycle delay at the G2/M-transition. This could be due to the beneficial influence of acetylation on microtubular stabilization [103]. HDIs also resulted in thymidylate synthase downregulation, an enzyme involved in the folate cycle and a goal of cytostatic agents such as pemetrexed. Pemetrexed resistance is associated with increased thymidylate synthase levels. Numerous NSCLC cell lines exhibited a synergistic effect on growth inhibition and apoptosis if treated consecutively with pemetrexed and ITF2357, a pan-HDI. The findings were verified in xenograft models obtained from the H1650 adenocarcinoma cell line [104]. The HDI romidepsin was able to improve erlotinib anti-tumour effect in nine NSCLC lines with varying histology and mutation conditions, including EGFR-, KRAS-mutant, and wild type cell lines, with a decrease in tumour load in NCI-H1299 xenograft models [105]. HDAC inhibition, plus entinostat (MS-275), re-sensitized TKI-refractory NSCLC cell lines to gefitinib, most likely by recovering E-cadherin expression [106].

Panobinostat, a pan-HDI, not only made TKI-resistant A549 cells susceptible to erlotinib’s antineoplastic action, but it also increased mono-, di-, and trimethylation of histone H3 lysine 4 (H3K4), indicating an intermodulation between HDI and LSD1 [107]. Moreover, panobinostat was able to prime NSCLC cell lines for the distinguishing effect of all-trans retinoic acid (ATRA) [108], and incorporating ATRA with the novel HDIs SL142 or SL325 repressed colony initiation, mediated apoptosis via Bax expression, and elevated caspase-3 activity in NSCLC cell lines [109]. The HDI (and anti-convulsant) sodium valproate also improved the anti-tumoural impact of cisplatinum-vinorelbine-based chemoradiation in NSCLC cell lines [110]. TSA also was able to radio sensitize NSCLC cell lines by initiating apoptosis and G2/M-cell-cycle arrest [111].

When combined with other agents, HDIs have a more pronounced overall impact. Vorinostat was shown to be effective in advanced lung cancer when coupled with carboplatin and paclitaxel [112]. In NSCLC cells, a combination of TSA and etoposide inflicted apoptotic cell death. Nutritional factors genistein and carotene, when combined with TSA, improved the cell growth arrest impact in A549 NSCLC cells [113]. The addition of low-dose vorinostat to 5-FU treatment increased drug-mediated cytotoxicity and contributed to synergistic implications, particularly in 5-FU-resistant NSCLC cells. Vorinostat could resolve 5-FU resistance by inhibiting thymidylate synthase expression and increasing p21waf1/cip1 expression through histone acetylation at its promoter. This was the first evidence that vorinostat increased 5-FU sensitivity in lung cancer cells by modulating 5-FU metabolism, and it will help future clinical trials of coupled chemotherapy and vorinostat in patients with NSCLC [114].

Millward et al. examined advanced solid tumours, including NSCLC cells, with vorinostat and marizomib, a novel bicyclic proteasome inhibitor, and discovered a heavily synergistic anti-tumour effect [115]. Despite the fact that no responses were observed using “Response Evaluation Criteria in Solid Tumours” (RECIST) criteria, over 60% of patients under evaluation had stable disease, with 39% showing reduction of the tumour size [115]. Combined therapy of vorinostat and arsenic trioxide enhances in vitro and in vivo H1299 NSCLC cell death [116]. Furthermore, combining suboptimal doses of sulindac, a non-steroidal anti-inflammatory drug, with vorinostat resulted in growth inhibition of A549 human NSCLC cells, primarily through increased mitochondrial membrane potential collapse, cytochrome c release, and caspase induction [117]. Table 1 summarizes some currently underway clinical trials for the treatment of lung cancer with hypomethylating agents and HDIs [15]. Furthermore, the modification of histone methylation has the potential to improve the efficacy of chemotherapeutic agents as a combination therapy in lung cancer. Protein arginine methyltransferase 5 (PRMT5), for example, is highly expressed in lung ADC and is implicated in tumourigenesis. In ADC, the combination of PRMT5 inhibitor, arginine methyltransferase inhibitor 1 (AMI-1), and cisplatin resulted in anti-tumour activity by substantially decreasing cell viability and initiating apoptosis. Furthermore, this combination had no effect on the survival of normal bronchial epithelial cells [118].

#### 3.3.2. Clinical Studies of Combined Therapies with Histone Modifiers

Jones et al. demonstrated that HDAC inhibition with vorinostat, coupled with proteasome inhibition with bortezomib preceded by surgical procedure, was a viable treatment strategy after performing a phase I study with 21 NSCLC patients [79]. Another phase I study investigated combined vorinostat with sorafenib (a multiple kinase inhibitor) in 17 patients with various cancers, including three with NSCLC, of whom one had a single case of grade V haemoptysis and another had a coronary incident. [80]. Panobinostat with erlotinib was well tolerated in a phase 1 trial in 33 patients with NSCLC, with dose-limiting toxicities emerging in two patients (nausea and prolonged QTc) that resolved without intervention. In a phase I/II study with 33 NSCLC EGFR-mutant patients who had progressed following erlotinib treatment, vorinostat combined with erlotinib only inhibited growth in 28 percent of the patients at 12 weeks following treatment [82].

In a phase II trial of 132 stage III and IV NSCLC patients who had progressed after pre-treatment (one or two previous chemotherapy or chemo-radiotherapy regimens), entinostat in combination with the TKI, erlotinib, had no benefit on the sample population. Yet, compared to erlotinib alone, patients with high E-cadherin levels at recruitment had a longer OS [71] Ramalingam et al. involved 94 patients with advanced NSCLC in a randomly selected, double-blind, placebo-controlled phase II trial evaluating cisplatin/paclitaxel combined with vorinostat or a placebo. The combination therapy was advantageous in terms of response rate, but not in terms of median progression-free survival (PFS) or OS [70]. Although results are limited by the occurrence of adverse effects, it is required to develop epigenetic modifiers with safer profiles and to select the subset of patients who may benefit from such a combination of therapies.

### 3.4. Combinatory Epigenetic Therapy

The discovery that HDACs play a role in TSG silencing in cancer supports the hypothesis that combining DNA methyltransferase (DNMT) and HDAC inhibition could result in improved or maintained TSG reactivation. This hypothesis is supported by preclinical data. Cameron et al. illustrated that the combination of decitabine and TSA reactivated silenced TSGs in colon and leukaemia cell lines [119], displaying that the functional correlation between DNA methylation and histone deacetylation in gene silencing might be manipulated pharmaceutically. Briefly after, in vitro studies revealed additive or synergistic inhibition of DNA production, loss of clonogenicity, and activation of apoptosis in NSCLC cell lines medicated with decitabine and an HDI (phenylbutyrate, depsipeptide, or TSA) [120]. Furthermore, combining decitabine and phenylbutyrate was shown to synergistically suppress the progression of lung lesions in mice after exposure to the carcinogen nicotine-derived nitrosamine ketone (NNK) [121].

A combination of azacitidine and entinostat was tried in patients with NSCLC, with some promising outcomes (phase I/II). Clinical efficacy was observed for a relatively long duration (one complete response for 14-month duration). In a search of a correlative biomarker, there was a higher response rate and improvement in OS in the “methylation signature”—positive patients compared to patients with a negative signature. Promotor methylation status was tested through liquid biopsy (circulating DNA) from patients on day 0 and day 29 (after one cycle of therapy) [85].

### 3.5. Combination Therapy of Histone Modifiers and Immune Checkpoint Inhibitors

Immune checkpoint inhibitors (ICIs) have emerged as promising agents for the treatment of several types of cancer. As they rely on the immunogenicity of the tumours, combining them with epigenetic modifiers is an active field of research. As previously demonstrated, a cross talk and overlap exist between methylation and histone modification in the context of NSCLC. Intriguingly, treatment of tumour cells with DNMT inhibitors can stimulate the transcription of the constitutively silenced endogenous retrovirus (ERVs) [122], thus forming double-stranded RNAs in the cytoplasm [123], the ligand of the retinoic acid inducible gene I-like receptors (RLR) [70,124]. The RLR family is considered a group of innate immune sensors, and its activation initiates signalling cascades leading to interferon production by CD8+ T cells, initiating an anti-tumour immune response [125]. Furthermore, epigenetic modifying agents mediate over-expression of tumour antigens (e.g., cancer testis antigens), increasing immunogenicity [126]. It is obvious that the combination of ICIs with epigenetic modifiers may be a promising strategy in the treatment of solid tumours.

In a recent study, several novel dual inhibitors of HDAC and heat-shock protein 90 (HSP90) were investigated in A549 and H1975 (NSCLC EGFR-resistant) cell lines. Interestingly, two of the tested compounds significantly reduce the PD-L1 expression of IFN-γ-treated H1975, referring to the promising role of histone modifiers in enhancing the effect of immunotherapy [127].

Combining HDIs with ICIs was investigated following a study that initially evaluated dual epigenetic modulation with entinostat and azacitidine, which did not result in a desirable anti-tumour response in patients with NSCLC. However, a patient subgroup proceeded to receive the anti-PD-1, nivolumab. Out of six patients, five showed a PFS of 6 months following the immunotherapy, a significant outcome in patients who had previously progressed on an ICI [85,128]. Epigenetic modification has primed the tumour to the action of the immunotherapy. An ongoing phase II trial is testing azacitidine and entinostat with concurrent nivolumab in patients with metastatic NSCLC, in immunotherapy-naïve versus immunotherapy-resistant patients (NCT01928576).

Furthermore, clinical trials of EZH2 inhibitors, combined with the PD-1 inhibitor “pembrolizumab” or the CTLA-4 inhibitor “ipilimumab”, are under trial in advanced solid tumours (NCT03854474 and NCT03525795). In addition, tumour-associated antigens expressed in NSCLC include melanoma-associated antigen A3 (MAGEA3) and mucinous glycoprotein-1 (MUC1). MAGEA3 and MUC1 vaccines have both shown a potential effect and are undergoing testing in phase II and phase III trials [129].

## 4. Conclusions and Future Direction

Despite the fact that epigenetic studies in cancer, including histone modifications, started a few decades ago, the clinical application of histone modifying agents is still in its early stages and is not yet fully endorsed by the results of large-scale clinical trials. However, the intricate aspects of the cancer epigenome have highlighted tumour heterogeneity and the fact that cancer cell mutations are, by far, not the only initiators and drivers of carcinogenesis, but instead, the effects of those mutations are tightly controlled and directed by the epigenetic signature, which in turn is affected by many environmental factors.

Epigenetic modifiers are unlikely to enter into clinical practice in medical oncology as a standalone monotherapy. Instead, such medications can be combined with classic conventional anti-cancer medications, targeted therapies, or immune checkpoint inhibitors. The concerted action of combined medications should be selected for specific patient subsets who are likely to benefit from such therapies. Clinical trials showed that the adverse effects of such combination therapies are critically affecting the therapeutic outcomes, despite the relatively safe profile of epigenetic modifiers. Further research is required to appropriately place the epigenetic modifiers in the treatment algorithm of non-small cell lung cancer.

## Figures and Tables

**Table 1:** Histone-modification-based epigenetic therapy in clinical trials for NSCLC.

Drug	Target/ Mechanism of Action	Phase of Trial	Number of Patients	Outcomes	Clinical Trial Identifier/ Reference
**Histone Deacetylase Inhibitors**					
Vorinostat	Prevents enzymatic activities of class I and II HDACs, elicits cell arrest, differentiation, and/or apoptosis, antiproliferative, G1/G2 cell cycle arrest, disrupts VEGF signalling	Phase I	2 NSCLC/73 patients	CR = 1, PR = 3, unconfirmed PR = 2, linear pharmacokinetics with good bioavailability	NCT00045006 [72]
		Phase II	16 patients	SD = 8, PR = 1, PD = 3	NCT00565227 [73]
		Phase II	8 patients	SD (3.7 months) = 8, OS = 7.1 months	NCT00126451 [74]
Romidepsin	Triggers p21 expression, H4 acetylation, shift gene signature to normal epithelia	Phase II	19 Lung Cancer patients	Transient SD = 9	NCT00020202 [75]
Pivanex	Induces tumour cell differentiation and/or apoptosis	Phase II	47 refractory NSCLC patients	PR = 6.4%, SD (>12 weeks) = 30%, MS = 6.2 months1 year survival rate = 26%	[76]
Cl-994	Inhibits histone deacetylation, G1-S phase cell arrest	Phase I	53 solid tumours	PR = 1 heavily-pre-treated NSCLC patient SD = 3 (1 NSCLC patient)	[77]
**Combination therapy**					
Vorinostat + Carboplatin /Paclitaxel	-	Phase I	28 advanced solid tumour patients	PR = 11 (10 NSCLC), SD = 7 Linear Pharmacokinetics	[78]
Vorinostat + Carboplatin/ Paclitaxel	Enhances the anti-cancer effects of platinum compounds and taxanes	Phase II	94 advanced (stage IIIB or IV NSCLC patients)	Enhanced response rate (34%) OS = 13 months	NCT01413750 [70]
Vorinostat + Bortezomib	Combined induction proteasome and histone deacetylase inhibition	Phase I	21 patients	Tumour necrosis (30%)	[79]
Vorinostat + Sorafenib	-	Phase I	17 patients with advanced solid tumours	Unconfirmed PR = 2 (1 NSCLC patient)	[80]
Vorinostat + Erlotinib	-	Phase I/II	33 advanced NSCLC EGFR mutant patients	PFS = 8 weeks OS = 10.3 months	NCT00503971 [81]
Panobinostat + Erlotinib	-	Phase I	35 NSCLC/42 patients with advanced tumours	Disease control rate = 54%, NSCLC PR =3, SD = 3 PFS = 4.7 months, OS = 41 months, (EGFR mutation)	NCT00738751 [82]
Entinostat + Erlotinib	-	Phase II	132 stage IIIB and IV NSCLC patients	Longer OS (9.4 months) in high E-cadherin patients	NCT00602030 [71]
Pivanex + Docetaxel	Synergistic action for growth inhibition of NSCLC cell lines in vitro and for improved survival in animal models	Phase I Phase IIb	12 patients 225 patients	Results not published	NCT00073385
Cl-994 + Gemcitabine	-	Phase II	26 NSCLC/174 patients	PR = 8, OR = 12%, MS = 194 days	NCT00005093 [83]
Cl-994 + Carboplatin + Paclitaxel	-	Phase I	30 patients with advanced solid tumours	H3 acetylation levels <1.5-fold times baseline = PD, H3 acetylation levels ≥1.5-fold times baseline = Clinical response/SD, PR = 5 (3 NSCLC)	[84]
Azacitidine + Entinostat	Inhibition of promoter methylation	I/II	45 advanced, refractory NSCLC	MS = 6.4 months, CR =1, PR = 1	NCT00387465 [85]
Decitabine + valproic acid	Inhibitors of DNA methylation and histone deacetylases	I	8 patients with advanced NSCLC with prior chemotherapy	SD = 1	NCT00084981 [86]
Decitabine + vorinostat	Inhibitors of DNA methylation and histone deacetylases	I	2 patients with NSCLC/44 with advanced tumours	SD = 29%	NCT00275080 [85]
Azacitidine + sodium phenylbutyrate	Inhibitors of DNA methylation and histone deacetylases	I	1 NSCLC/27 refractory Solid Tumours	SD = 1, PD = 26	NCT00005639 [87]
Hydralazine+ magnesium valproate	Reduction in global DNA methylation, histone deacetylase activity, and promoter demethylation were observed	II	1 NSCLC/17 refractory solid tumours	PR = 4, SD = 8	NCT00404508 [88]

CR: complete response, PR: partial response, SD: stable disease, PD: progressive disease, MS: median survival, OS: overall survival, PFS: progression-free survival.

## Data Availability

Not applicable.

## Abbreviations

AC	Adenocarcinoma
ATRA	All-Trans Retinoic Acid
AMI-1	Arginine Methyltransferase Inhibitor 1
ATO	Arsenic Trioxide
AZA	Azathioprine
CO	Carbon Monoxide
CR	Complete Response
COX-2	Cyclooxygenase-2
DAPK	Death-Associated Protein Kinase
DNA DSB	DNA Double Strands Break
DNMT	DNA Methyltransferase
EGFR	Epidermal Growth Factor Receptor
FDA	Food and Drug Administration
HDI(s)	HDAC Inhibitors
HATs	Histone Acetyltransferases
HDACs	Histone Deacetylases
HDI	Histone Deacetylases Inhibitors
HDMs	Histone Demethylases
KDMs	Histone Lysine Demethylases
KMTs	Histone Lysine Methyltransferases
HMTs	Histone Methyltransferases
HLA	Human Leukocyte Antigen
ICI	Immune Checkpoint Inhibitor
IL	Interleukins
IARC	International Agency for Research on Cancer
ICAM-1	Intracellular Adhesion Molecule-1
LCC	Large Cell Cancer
lncRNAs	Long Non-Coding RNAs
LSD1	Lysine Specific Demethylase
MAGEA3	Melanoma-Associated Antigen A3
MCP-1	Monocyte Chemotactic Protein-1
MDS	Myelodysplastic Syndromes
MHC	Major Histocompatibility Complex
MICA	MHC Class I Chain-Related Molecules
mRNA	Micro RNA
MUC1	Mucinous Glycoprotein-1
NNK	Nicotine-Derived Nitrosamine Ketone
NOx	Nitrogen Oxides
ncRNAs	Non-Coding RNAs
NSCLC	Non-Small Cell Lung Carcinoma
PR	Partial Response
PM	Particulate Matter
P2RD	Phase 2 Suitable Dose
PTMs	Post-Translational Modifications
PFS	Progression-Free Survival
PRMT5	Protein Arginine Methyltransferase 5
ROS	Reactive Oxygen Species
RFS	Relapse-Free Survival
RECIST	Response Evaluation Criteria in Solid Tumours
SCLC	Small Cell Lung Cancer
SCC	Squamous Cell Carcinoma
SAHA/Vorinostat	Suberoylanilide Hydroxamic Acid
SO_2_	Sulphur Dioxide
TSA	Trichostatin A
TSG	Tumour Suppression Genes
TKI	Tyrosine Kinase Inhibitor
VEGF	Vascular Endothelial Growth Factor
abb	ddd
AC	Adenocarcinoma
